# The Effect of MicroRNA-124 Overexpression on Anti-Tumor Drug Sensitivity

**DOI:** 10.1371/journal.pone.0128472

**Published:** 2015-06-26

**Authors:** Shiau-Mei Chen, Wen-Cheng Chou, Ling-Yueh Hu, Chia-Ni Hsiung, Hou-Wei Chu, Yuan-Ling Huang, Huan-Ming Hsu, Jyh-Cherng Yu, Chen-Yang Shen

**Affiliations:** 1 Graduate Institute of Life Sciences, National Defense Medical Center, Taipei, Taiwan; 2 Institute of Biomedical Sciences, Academia Sinica, Taipei, Taiwan; 3 Department of Surgery, Tri-Service General Hospital, Taipei, Taiwan; 4 College of Public Health, China Medical University, Taichong, Taiwan; Duke University, UNITED STATES

## Abstract

MicroRNAs play critical roles in regulating various physiological processes, including growth and development. Previous studies have shown that microRNA-124 (miR-124) participates not only in regulation of early neurogenesis but also in suppression of tumorigenesis. In the present study, we found that overexpression of miR-124 was associated with reduced DNA repair capacity in cultured cancer cells and increased sensitivity of cells to DNA-damaging anti-tumor drugs, specifically those that cause the formation of DNA strand-breaks (SBs). We then examined which DNA repair–related genes, particularly the genes of SB repair, were regulated by miR-124. Two SB repair–related genes, encoding ATM interactor (ATMIN) and poly (ADP-ribose) polymerase 1 (PARP1), were strongly affected by miR-124 overexpression, by binding of miR-124 to the 3¢-untranslated region of their mRNAs. As a result, the capacity of cells to repair DNA SBs, such as those resulting from homologous recombination, was significantly reduced upon miR-124 overexpression. A particularly important therapeutic implication of this finding is that overexpression of miR-124 enhanced cell sensitivity to multiple DNA-damaging agents via ATMIN- and PARP1-mediated mechanisms. The translational relevance of this role of miR-124 in anti-tumor drug sensitivity is suggested by the finding that increased miR-124 expression correlates with better breast cancer prognosis, specifically in patients receiving chemotherapy. These findings suggest that miR-124 could potentially be used as a therapeutic agent to improve the efficacy of chemotherapy with DNA-damaging agents.

## Introduction

MicroRNAs are noncoding small RNAs that contribute to the regulation of their cognate target genes, usually by imperfect base-pairing with the 3′-untranslated region (UTR) of the target mRNA, which results in cleavage/degradation of the mRNA and translational repression [1]. MicroRNAs play critical roles in regulating various physiological processes, including growth and development, and thus microRNA abnormalities are often involved in the initiation and progression of cancer [2]; indeed, microRNA expression profiling indicates that microRNAs can function as oncogenes or tumor suppressors [3]. Because of their potential to regulate a large number of protein-encoding genes, microRNAs are also a promising new target in the development of clinical treatments [4]. MicroRNA-124 (miR-124) is enriched in the brain and promotes neuronal differentiation [5]. Interestingly, miR-124 also plays a key role in cancer cell proliferation and is epigenetically silenced in various types of cancer [6, 7]. Selected examples include (a) miR-124 modulates cell growth via regulating the expression of cyclin-dependent kinase 6 [6]; (b) miR-124 suppresses hepatocellular carcinogenesis by inducing tumor-specific apoptosis [8]; (c) miR-124 suppresses invasion and migration of oral squamous cell carcinoma by downregulating ITGB1 expression [9]; (d) the expression of phosphoinositide 3-kinase catalytic subunit alpha can be suppressed by miR-124, resulting in suppression of PI3K/Akt pathway and proliferation of hepatocellular carcinoma [10]; (e) miR-124 affects proliferation and motility of cancer cells by repressing ROCK2 and EZH2 [11]; (f) miR-124 determines the epithelial phenotype of breast cancer cells, by targeting the epithelial–mesenchymal transition regulator Slug and increasing the expression of E-cadherin, a hallmark of epithelial cells [12]. All these findings suggest that miR-124 plays a crucial role as tumor suppressor in different kinds of tumors. Such dysregulated microRNA expression can result from aberrant DNA methylation and has been observed in cancer cell lines of different tissue origins, including colon, breast, lung, stomach, cervical, and liver [13–15]. However, these aspects have not been examined in detail in actual cancers. For example, miR-124 is downregulated by hypermethylation of its promoter in the breast cancer cell line MDA-MB-231 [14], but, to the best of our knowledge, its methylation and expression have not been examined in tumors from breast cancer patients. In our recent genome-wide association analysis of genomic loci associated with lymph node metastasis of breast cancer, genetic polymorphism of the locus harboring miR-124 was among the top loci determining the metastatic phenotype [16], providing genetic evidence to support the idea that miR-124 dysregulation makes a critical contribution to cancer progression in patients. The important role of miR-124 in regulating cancer metastasis and the abnormal expression of miR-124 detected during cancer progression prompted us to explore whether miR-124 could be a therapeutic agent or could affect treatment efficacy. Toward this end, we examined the effects of modulating miR-124 expression on cellular responses to the drugs used in cancer chemotherapy.

The present study addresses the question regarding the role of miR-124 in the treatment of breast cancer and osteosarcoma. Breast cancer is the leading cause of cancer incidence in females in many populations and countries [17]. To treat this disease, the expression of estrogen receptor (ER), progesterone receptor (PR) and Her2 determines the choice of therapy regimens as well as predicts progression of patients. In different subtypes of breast cancer, the triple negative subtype, defined as no/few expression of ER, PR and Her2 and usually harboring *TP53* and *BRCA*1 mutation, comprises 10–20% of all breast cancers, and are at high risk of tumor recurrence due to the lack of reliable specific target of chemotherapy [18]. On the other hand, osteosarcoma is the second leading cause of cancer-related death in early-onset cancers, mainly due to development of metastasis [19]. Eighty percent of osteosarcoma patients are treated with multi-agent chemotherapy in addition to surgical resection [20]. Similar to that in the triple-negative breast cancer, reliable and effective chemotherapeutic target for osteosarcoma has remained unavailable.

Disruption of DNA repair is a common approach used in many current cancer therapies. Inhibition of specific DNA repair pathways is more efficacious when combined with DNA-damaging chemotherapeutic drugs [21]. One of the more promising targets, which has attracted wide attention, is the DNA double-strand break-repair protein poly (ADP-ribose) polymerase 1 (PARP1). PARP1 inhibitors sensitize tumor cells to cytotoxic drugs such as the alkylating agent temozolomide (TMZ) and cyclophosphamide and the topoisomerase I inhibitors irinotecan and topotecan [22, 23]. Moreover, ataxia telangiectasia mutated (ATM) is an important DNA damage checkpoint kinase, and its interactor protein, ATMIN, regulates its activity via competition with the DNA double-strand break–associated factor nibrin [24–26]. ATMIN deficiency enhances ionizing radiation (IR)-induced ATM signaling and the development of B-cell lymphomas [26, 27]. Here we demonstrate that miR-124 directly targets the mRNAs encoding both ATMIN and PARP1, suggesting that miR-124 may constitute a target or potential therapeutic in cancer treatment.

## Materials and Methods

### Cell lines and culture

All cells were purchased from the Taiwan Bioresource Collection and Research Center (Hsinchu, Taiwan). The MDA-MB-231, HCC1937, T-47D and ZR-75-1 were cultured in RPMI-1640 medium (Sigma-Aldrich, St. Louis, MO) with 10% fetal bovine serum (Gibco, Grand Island, NY). The BT474 was cultured in 90% Hybri-Care Medium (Sigma-Aldrich, St. Louis, MO) with 30 ng/ml EGF and 10% fetal bovine serum. The MDA-MB-453 was cultured in 90% Leibovitz's L-15 medium (Sigma-Aldrich, St. Louis, MO) with 10% fetal bovine serum and without CO_2_. The MCF-7 and Hs 578T were cultured in Dulbecco's Modified Eagle Medium (Sigma-Aldrich, St. Louis, MO) with 10% fetal bovine serum. The MDA-MB-361 was cultured in 80% Leibovitz's L-15 medium with 20% fetal bovine serum and without CO_2_. The U-2 OS was cultured in McCoy’s 5A medium (Sigma-Aldrich, St. Louis, MO) with 10% fetal bovine serum.

### Real-time reverse transcription-PCR

The mRNA was extracted from cultured cells using TRIZOL reagent (Invitrogen, Carlsbad, CA) and reverse transcribed with SuperScript III reverse transcriptase (Invitrogen). MiR-124 levels were determined using the TaqMan miRNA assay (Applied Biosystems, Foster City, CA) and quantified using the comparative CT method [19] and normalized to RNU6B.

### Clonogenic survival assay

Long-term survival was determined by a colony-forming assay. A total of 500 cells were plated in each well of a six-well plate and incubated at 37°C. After 24 h, cells were treated with camptothecin (CPT; 0, 5, 10, or 20 nM), etoposide (ETO; 0, 5, 25, or 50 nM), doxorubicin (DOX; 0, 5, 10, or 20 nM), IR (0, 4, 8, or 10 Gy), TMZ (0, 10, 50 or 100 μM) or 5-fluorouracil (5-FU; 0, 10, 50, or 100 μM). Cell monolayers were rinsed twice with phosphate-buffered saline (PBS) and then resupplied with fresh medium. After 10 days, colonies were stained with 0.1% (w/v) crystal violet solution and counted to determine the surviving fractions. Experiments were performed in triplicate.

### MiR-124 target prediction

Potential direct miR-124 targets were predicted using the PicTar (http://pictar.mdc-berlin.de), TargetScan 5.1 (http://www.targetscan.org) and miRanda (http://www.microrna.org) algorithms.

### Luciferase reporter assay

The candidate miR-124 target-luciferase constructs and pRL-tk, which encodes Renilla luciferase, were co-transfected into 1 × 10^5^ cells in each well of a 24-well plate. After 48 h, the cells were lysed by freeze-thawing in passive lysis buffer, and then luciferase activity in the lysate was measured using the Dual-Luciferase Reporter Assay System kit (Promega, Madison, WI)[28].

### Cell lysis and western blotting

Preparation of whole-cell extracts and western blotting were performed as described [29]. Protein concentrations were determined using the Bio-Rad Protein Assay (Bio-Rad, Richmond, CA). The antibodies used for western blotting were rabbit polyclonal anti-ATMIN (A303-399A; Bethyl Laboratories, Inc., Montgomery, TX) and anti-α-tubulin (T6199; Sigma-Aldrich) and mouse monoclonal anti-PARP (GTX30110; Genetex, San Antonio, TX). Signals were detected using chemiluminescent reagents (Millipore, Temecula, CA).

### Comet assay

The alkaline comet assay was performed as described [29] using a CometAssay kit (4250-050-K; Trevigen, Gaithersburg, MD). The cells were seeded in six-well plates and incubated overnight at 37°C and then treated with 12 μM CPT for 1 h to induce DNA damage, followed by incubation in fresh medium for various times to allow DNA repair. To determine tail moment values—as an index of DNA damage—the cells were stained with YOYO-1 iodide (Invitrogen; 1:1000 dilution), visualized by fluorescence microscopy, and >100 cells per sample were analyzed by CometScore (TriTek Corp., Rancho Santa Margarita, CA).

### Cell viability assay

The viability and proliferation of MDA-MB-231 cells stably expressing miR-124 and transfected with the ATMIN or PARP1 expression plasmid were determined by 3-(4,5-dimethylthiazolyl-2-yl)-2-5 diphenyl tetrazolium bromide (MTT; Sigma-Aldrich) assay. The cells were plated in 24-well plates (1 × 10^4^ cells per well) in a final volume of 500 μL of medium and transfected with the ATMIN or PARP1 expression plasmid. After incubation for 96 h at 37°C, the culture medium was replaced with fresh medium. MTT stock solution (50 μL; 5 g/L in PBS) was added to each well to achieve a final concentration of 0.5 g/L. Plates were incubated for another 3 h at 37°C, then the culture medium was replaced with dimethyl sulfoxide (Sigma-Aldrich), and the absorbance was measured at 570 nm in a microplate reader. Cell viability was normalized to that of cells incubated in culture medium without treatment with CPT or ETO. Three independent experiments were performed with three replicates in each.

### Immunofluorescence staining

MDA-MB-231 cells stably transfected with the miR-124 expression or control vector were seeded on coverslips, fixed for 15 min at room temperature in 3.7% paraformaldehyde in PBS, washed three times with cold PBS, and permeabilized for 10 min at room temperature in PBS containing 0.1% (v/v) Triton X-100. The cells were blocked in PBS containing 5% bovine serum albumin then labeled for 30 min at room temperature with mouse monoclonal anti-γ- H2AX (Ser139; 05–636; Millipore, Billerica, MA) and goat anti-mouse fluorescein isothiocyanate (Invitrogen). Nuclei were stained with 4', 6-diamidino-2-phenylindole. The coverslips were then mounted on microscope slides with an antifade reagent (VECTASHIELD Mounting Media; Vector Laboratories, Inc., Burlingame, CA) and images taken using an LSM700 confocal microscope (Oberkochen, Germany). At least 100 cells were assessed per coverslip, and cells were considered positive for γ-H2AX if they had >5 foci per nucleus.

### Statistical analysis

All data are presented as means ± standard deviation (SD). Two sample *t* test and one-way analysis of variance (ANOVA) test were used to analyze the difference of measurements. The Kaplan–Meier plot was used to test the association between miR-124 expression and overall survival of patients. The p-values threshold of statistical significance was determined as 0.05.

## Results

### Downregulation of miR-124 in breast cancer and osteosarcoma cell lines

**Fig 1:**
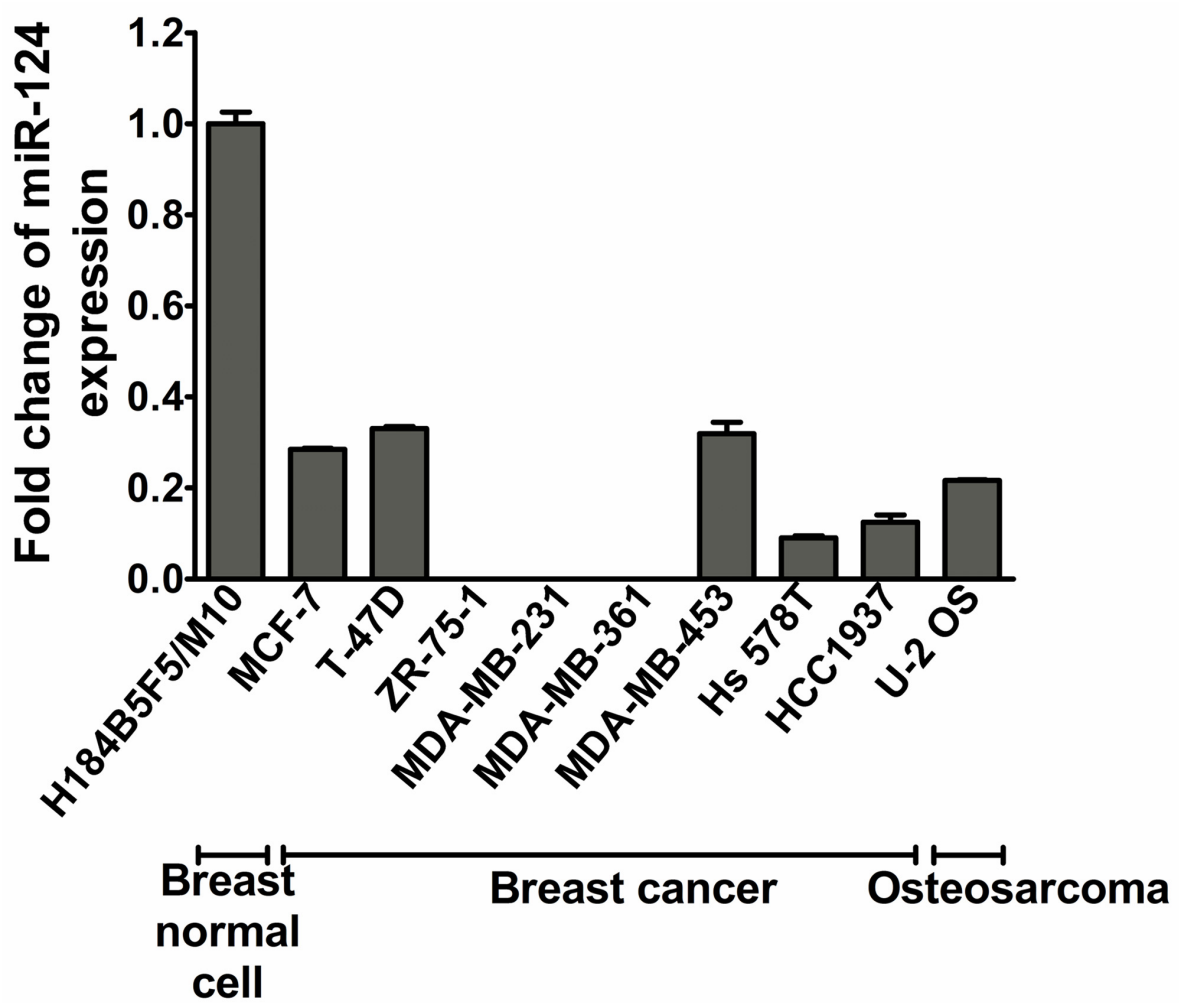
Expression of miR-124 mRNA in breast cancer cell lines and U-2 OS (osteosarcoma) cells. MiR-124 expression level was determined by quantitative PCR and expressed as the fold change relative to RNU6B. Expression in the immortalized breast epithelial cell line, H184B5F5/M10, was used as the reference.

To demonstrate the tumorigenic relevance of miR-124, we first examined miR-124 expression in eight breast cancer cell lines and an osteosarcoma cell line (U-2 OS) using stem-loop quantitative reverse transcription PCR. The levels of miR-124 in all nine lines were significantly reduced, to varying degrees, compared with H184B5F5/M10, an immortalized breast cell line (Fig 1). Notably, the triple-negative breast cancer cell line MB-MDA-231 had dramatically less expression of miR-124 than H184B5F5/M10.

### MiR-124 overexpression increases the sensitivity of breast cancer and osteosarcoma cells to anti-tumor drugs

**Fig 2:**
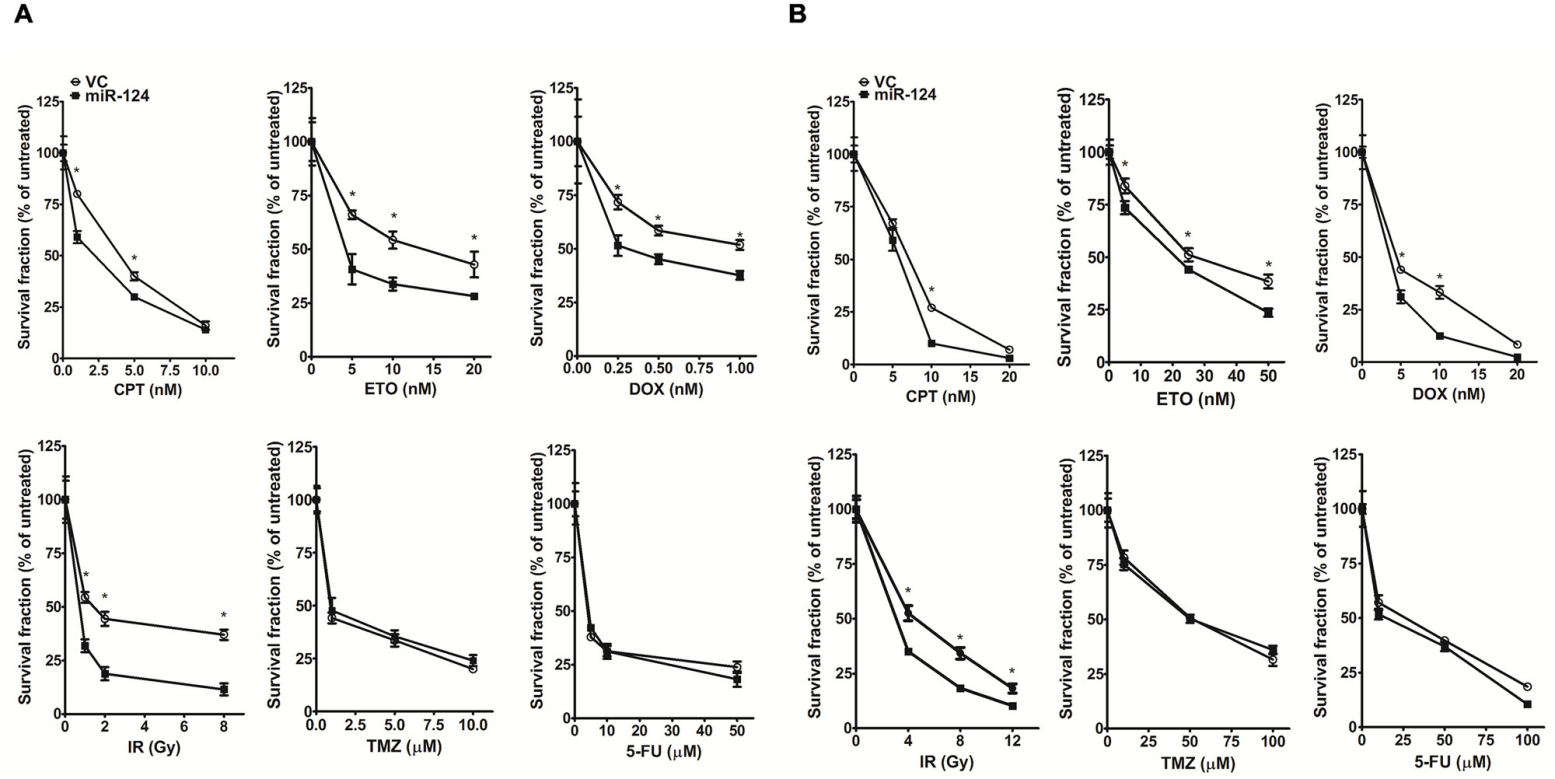
MiR-124 overexpression increases sensitivity to specific anti-tumor drugs/treatments. (A) Survival of breast cancer cells (MDA-MB-231) stably transfected with the empty lentivirus vector (vector control, VC) or the miR-124-overexpressing construct (miR-124) after the indicated treatments. (B) Survival of osteosarcoma cells (U-2 OS) stably transfected with the empty lentivirus vector (vector control, VC) or the miR-124-overexpressing construct (miR-124) after the indicated treatments. *Significant difference (*P* < 0.05) between the miR-124 and VC-transfected cells.

To assess the effects of miR-124 expression on drug sensitivity, we constructed a lentiviral miR-124 overexpression vector for use in an MB-MDA-231 cell survival assay. To determine the efficiency of miR-124 on regulation of cellular response to anti-cancer drugs, we used both transient transfection, transfecting different dosages of miR-124 into MDA-MB-231 cells, and stable transfection, in which the clones expressing high and low levels of miR-124 were selected for experiments. The results show that higher level of miR-124 expression resulted in higher sensitivity of cell to drugs (S1 Fig). Based on this, we examined six common anti-tumor drugs/treatments, including CPT, ETO, DOX, IR, TMZ, and 5-FU. Cell death increased more in the miR-124-overexpressing cells than in the vector control group after CPT, ETO, DOX, or IR treatment (Fig 2A and Fig. A in S2 Fig). However, the effect of miR-124 seemed to be specific to the type of damage because we did not observe any differences between the miR-124-overexpressing and vector control groups in the TMZ or 5-FU treatments (Fig 2A). These findings suggested that miR-124 overexpression increased sensitivity to drugs/treatments, especially those that induce DNA damage.

We next examined whether miR-124 overexpression has the same effect on drug sensitivity of the osteosarcoma cells (U-2 OS cell). The U-2 OS cells were derived from osteosarcoma, and commonly used in the investigation of the mechanisms of DNA repair and response. This is because that this cell carries wild–type *TP53* and *BRCA1*, preventing significant confounding effect due to loss of *TP53* and *BRCA1* [30, 31]. As in the breast cancer cell line, miR-124 overexpression increased the sensitivity of U-2 OS cells to each of CPT, ETO, DOX, and IR but not to TMZ or 5-FU (Fig 2B and Fig. B in S2 Fig).

### MiR-124 regulates the DNA repair–related genes ATMIN and PARP1

**Fig 3:**
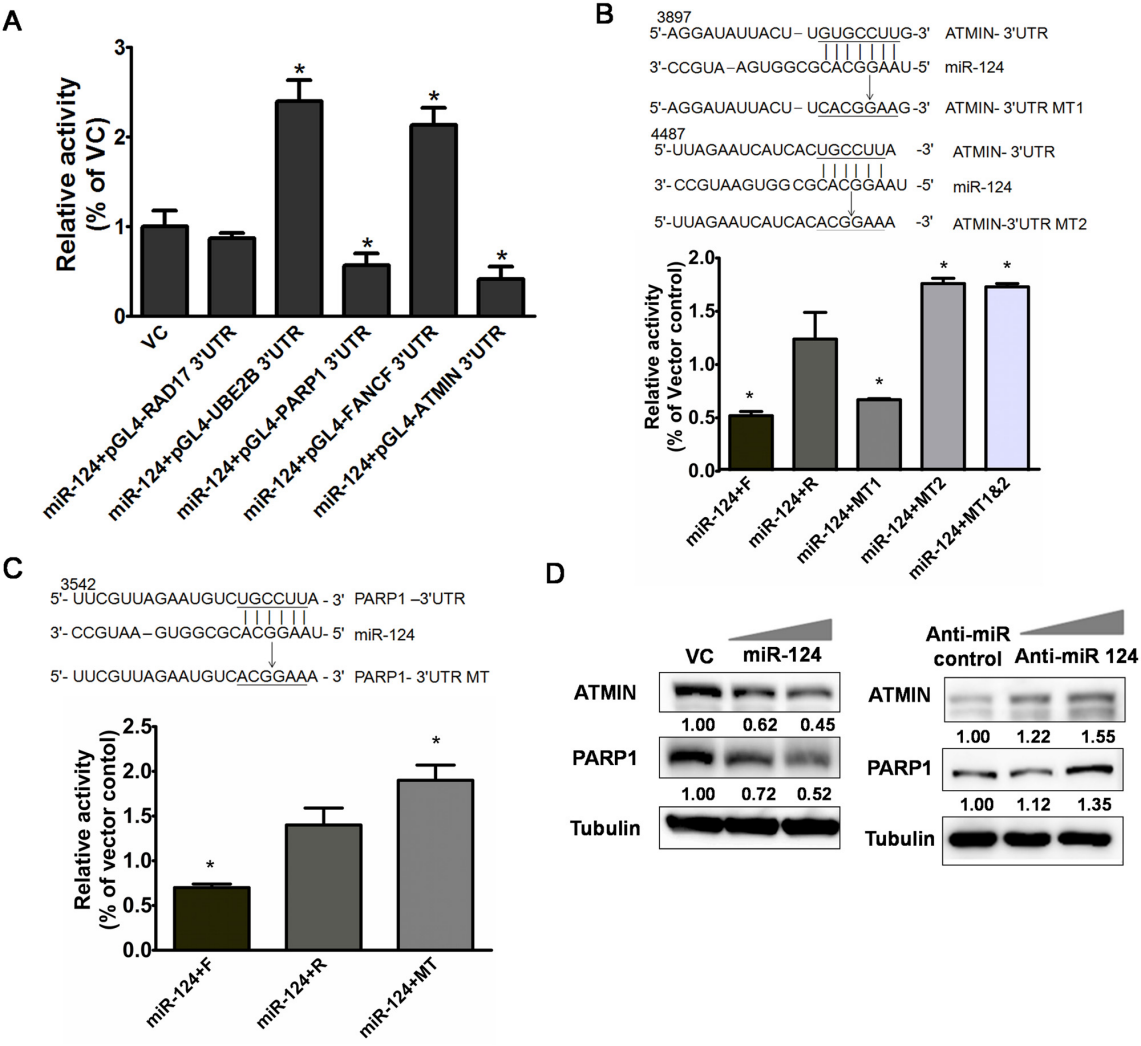
ATMIN and PARP1 mRNAs are targets of miR-124. (A) Relative luciferase activity in U-2 OS cells carrying the luciferase reporter (pGL4) linked to the sense-oriented (normally orientated) 3′-UTR of putative miR-124 targets *RAD17*, *UBE2B*, *PARP1*, *FANCF*, and *ATMIN* or the empty reporter vector control (VC). *Significantly different (*P* < 0.05) from the VC group. (B) Two predicted miR-124 binding sequences in *ATMIN-3*′-UTR (upper panel, ATMIN-3′-UTR) were mutated (upper panel, ATMIN-3′-UTR MT1 and ATMIN-3′-UTR MT2). (Lower panel) Expression of the reporter gene in U-2 OS cells when linked to the sense-oriented (normally oriented) (forward) *ATMIN*-3′-UTR (miR-124-F), reverse-oriented *ATMIN*-3′-UTR (miR-124-R), ATMIN-3′-UTR MT1 (miR-124-MT1), ATMIN-3′-UTR MT2 (miR-124-MT2), or the mutant carrying mutations at both of the two predicted sites (miR-124-MT1&MT2). (C) One predicted miR-124 binding sequence in *PARP1*-3′-UTR (upper panel, PARP1-3'-UTR) was mutated (upper panel, PARP1-3'-UTR MT). (Lower panel) Expression of the reporter gene in U-2 OS cells when linked to the [sense-oriented (normally oriented) (forward) PARP1-3'-UTR (miR-124-F), reverse-oriented PARP1-3'-UTR (miR-124-R, or PARP1-3'-UTR MT (miR-124-MT). (D) MiR-124 regulates expression ATMIN and PARP1. Western blot of ATMIN and PARP1 in U-2 OS cells transiently transfected with the VC or miR-124-expressing vector (miR-124; upper panel), or with a 2′-O-methyl-modified antisense inhibitor of miR-124 (Anti-miR-124; lower panel) or 2′-O-methyl negative control (Anti-miR) [48]. Tubulin was included as a protein loading control, and the numbers indicate the expression relative to expression in the VC or Anti-miR control, measured by densitometry. *P < 0.05 relative to VC (A) or to miR-124-R (B and C).

The damage type–specific effect of miR-124 that we detected prompted us to propose that miR-124 may target the genes involved in specific DNA repair pathways. Because CPT, ETO, DOX, and IR specifically cause formation of DNA strand-breaks (SBs) and the sensitivity of cancer cells to anti-tumor drugs is related to the expression of DNA repair genes [21], we were particularly interested in genes involved in DNA SB repair. To investigate potential targets of miR-124 regulation, we used the microRNA target prediction bioinformatics platforms, miRanda, TargetScan, and PicTar [32, 33]. We selected those potential target genes consistently predicted by these three platforms, resulting in 427 genes that may be the target of miR-124. To focus the genes specifically having a role in DNA repair, we used the Ingenuity Pathway Analysis [34] to specify the functions of these genes, and 81 of them were involved in DNA repair. We thoroughly and individually checked these functions, and included those only playing a direct role in DNA repair, concluding the five most likely genes, *Rad17*, *UBE2B*, *PARP1*, *FANCF*, and *ATMIN*. These five were examined by the following reporter assay. The wild–type full-length 3′-UTR of these genes was cloned into a firefly luciferase reporter vector. U-2 OS cells were transiently transfected with these constructs and the miR-124 overexpression vector. MiR-124 overexpression significantly suppressed the luciferase activity of the constructs containing the 3′-UTR of *ATMIN* and *PARP1* relative to the empty expression vector control (Fig 3A). We then introduced mutations in the two predicted miR-124 binding sites in *ATMIN*-3′-UTR and the one predicted binding site in *PARP1*-3′-UTR, and we examined the effects of these mutations on the response to miR-124 overexpression in the U-2 OS cell luciferase reporter assay (Fig 3B). We found that the reporter gene activity in miR-124-overexpressing cells transfected with the construct containing the reversed *ATMIN*-3′-UTR was significantly greater than that in the cells transfected with the wild-type forward *ATMIN*-3′-UTR reporter or the empty reporter plasmid. Mutation of the second, but not the first, putative miR-124 binding site in *ATMIN*-3′-UTR disrupted miR-124 overexpression–induced repression, indicating that miR-124 regulates *ATMIN* by binding to the second site (Fig 3B). Similarly, mutation of the predicted miR-124 binding site in *PARP1*-3′-UTR increased luciferase reporter gene activity, confirming that the 3'-UTR of *PARP1* is a binding target of miR-124 (Fig 3C). To examine whether these findings held true *in vivo*, we verified that ATMIN and PARP1 protein levels were affected by miR-124. Protein levels in U-2 OS cells transfected with a 2′-O-methyl-modified antisense inhibitor of miR-124 or 2′-O-methyl-negative control were examined by western blotting. With increased miR-124 expression, ATMIN and PARP1 levels substantially decreased. Conversely, inhibition of endogenous miR-124 resulted in upregulation of ATMIN and PARP1 (Fig 3D).

### Effects of miR-124 on DNA SB repair

**Fig 4:**
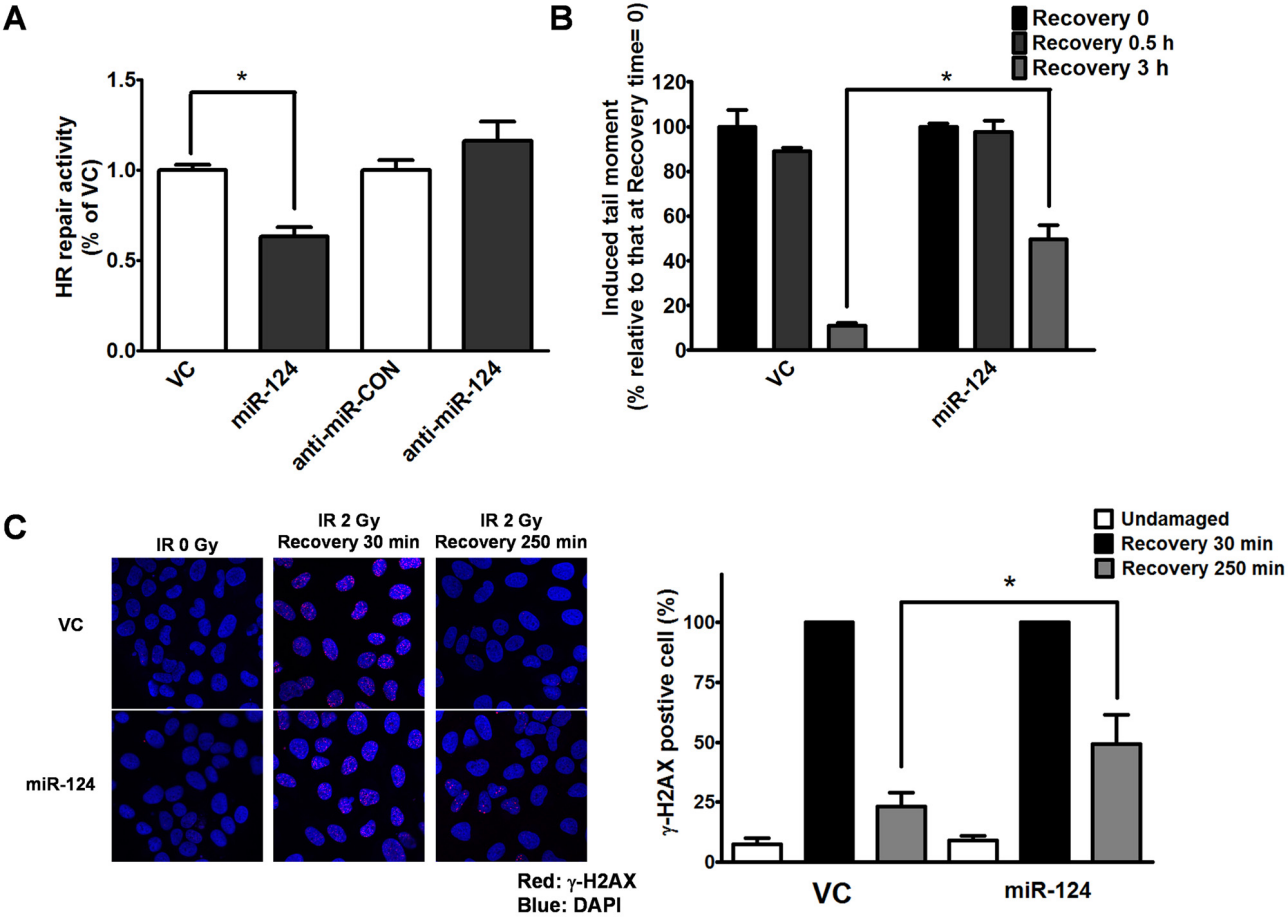
MiR-124 overexpression reduces DNA SB repair capacity. (A) HR repair capacity in miR-124-overexpressing cells and cells transfected with the antisense microRNA control (anti-miR-CON) or antisense miR-124 inhibitor (anti-miR-124). (B) Comet assay showing that miR-124 overexpression (miR-124) reduces SB repair relative to the vector control (VC) after treatment with CPT. (C) Immunostaining of DNA SBs (detected by the presence of γ-H2AX) in cells after IR treatment. Red: anti-γ-H2AX; blue: nuclei (left panel). The γ-H2AX signal was quantified and compared with the cells transfected with VC or miR-124 (right panel). All of the experiments shown were conducted in U-2 OS cells, and similar results were obtained in MDA-MB-231 cells. *P < 0.05 in individual comparisons.

The identification of ATMIN and PARP1 as potential targets of miR-124 prompted us to further test the effects of miR-124 on DNA SB repair. We thus examined homologous recombination (HR) repair—the pathway particularly important in repairing SBs—in cells using an established system based on strand exchange between two differently mutated enhanced green fluorescent protein gene sequences [35]. In this system, miR-124 overexpression reduced HR repair capacity to 40% of the vector control, whereas inhibition of endogenous miR-124 had no significant effect on HR repair (Fig 4A). We next performed a single-cell gel electrophoresis (comet) assay to further examine the extent of DNA damage/repair in cells overexpressing miR-124 after treatment with CPT for 1 h (S3 Fig). Cells overexpressing miR-124 had significantly different comet profiles than the vector control group, indicating that more DNA SB damage remained and, hence, a lower repair capacity, especially in the 3 h recovery from CPT group (Fig 4B). Because histone H2AX phosphorylation is a well-known marker of DNA SBs, we used immunostaining of phosphorylated H2AX (γ-H2AX) to examine the distribution of DNA damage after IR treatment. The miR-124-overexpressing and the vector control groups had similar proportions of γ-H2AX-positive cells after 30 min recovery from IR, but the miR-124-overexpressing cells appeared to have more γ-H2AX foci per cell than the vector control cells, suggesting more DNA damage in the miR-124-overexpressing cells (Fig 4C).

### Restoration of ATMIN and PARP1 expression reverses the DNA repair defect induced by miR-124 overexpression and increases resistance to anti-tumor drugs

**Fig 5:**
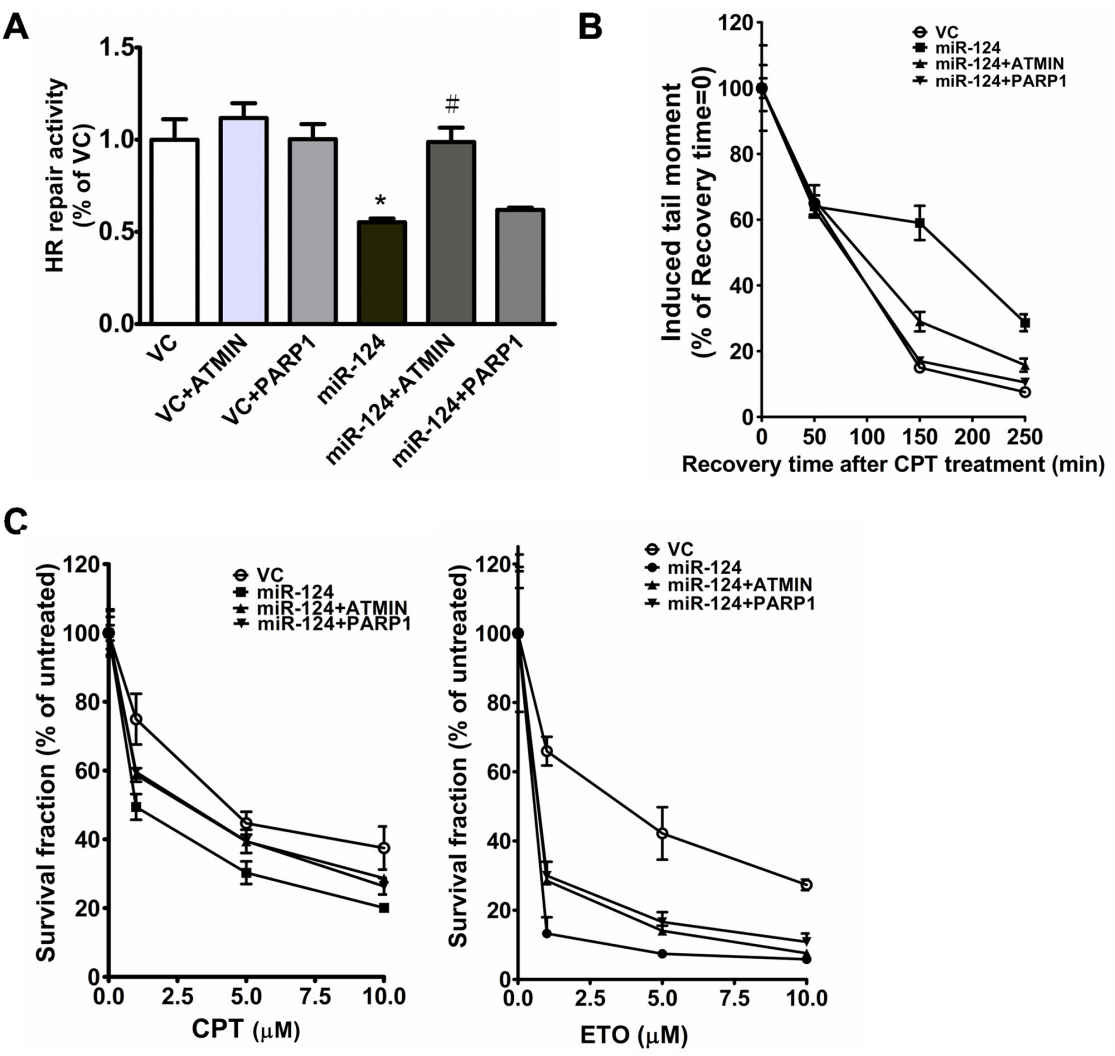
Introduction of ATMIN and PARP1 reverses the DNA repair defect induced by miR-124 overexpression. (A) HR repair capacity in cells transfected with the indicated combinations of vector control (VC) or the constructs overexpressing miR-124, ATMIN, or PARP1. *Significantly different (*P* < 0.05) from VC. ^#^Significantly different (*P* < 0.05) from the miR-124-overexpressing group. (B) Comet assay to detect DNA SBs in cells overexpressing VC, miR-124, miR-124 + ATMIN, or miR-124 + PARP1 after treatment with CPT. (C) Effects of ATMIN and PARP1 overexpression on miR-124 overexpression–enhanced sensitivity to CPT (left panel) or ETO (right panel). *Significantly different (*P* < 0.05) from VC.

To further explore whether miR-124 exerts its effects on DNA repair by downregulating the levels of mRNAs encoding ATMIN and PARP1, we restored ATMIN and PARP1 levels in a cell line stably expressing miR-124 and assessed the DNA repair capacity. In the HR assay, transient transfection with the ATMIN expression vector significantly increased DNA repair capacity in the miR-124-overexpressing cells to the level of the vector control. Restoration of PARP1 only slightly increased repair capacity compared with the miR-124-overexpressing group (Fig 5A and S4 Fig). This may be partially explained by that, as compared to ATMIN, PARP1 plays a relatively minor role of homologous recombination, which is the parameter measured in this HR assay. However, in the comet assay, restoration of PARP1 as well as ATMIN in miR-124-overexpressing cells decreased residual DNA damage after CPT treatment (Fig 5B). Next, we investigated whether restoration of ATMIN and PARP1 affected cell survival 5 days after CPT treatment. We found that increasing ATMIN or PARP1 equally increased cell survival in the miR-124-overexpressing groups. Similar increases in cell survival were seen when the levels of ATMIN and PARP1 were restored in miR-124-overexpressing ETO-treated cells (Fig 5C).

### Higher miR-124 level correlates with better cancer prognosis in patients receiving chemotherapy

**Fig 6:**
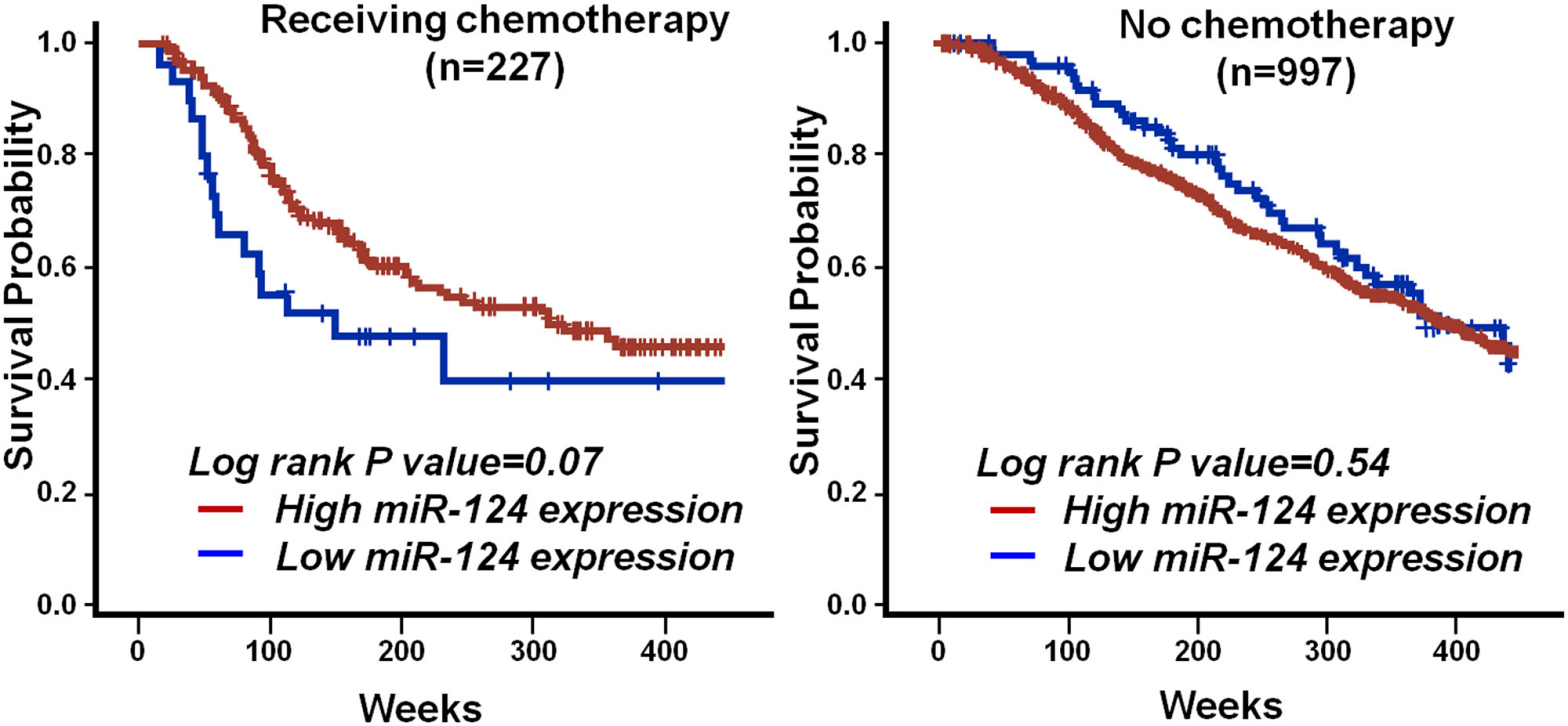
Higher miR-124 expression correlates with better prognosis for breast cancer patients receiving chemotherapy. Overall survival of breast cancer patients who received (left panel) or did not receive chemotherapy (right panel) stratified by expression of miR-124. Log-rank test *P* values are shown. Data were generated using the Kaplan Meier plotter [16].

We demonstrated that miR-124 overexpression reduced DNA SB repair, which may increase sensitivity to SB-inducing chemotherapeutic agents. The translational relevance of this finding was then examined by analysis of publically available data from a large breast cancer patient cohort study [16, 36]. The measurement of micro-RNAs expression in this cohort was based on chip-based experiments to detect expression in tumor tissues. We analyzed the association between overall survival and miR-124 expression level by the Kaplan-Meier plot. To this end, as many other studies using this dataset, we identified the median level of miR-124 expression in all patients, and defined those with higher/lower miR-124 expression using the median level as a cutoff [37]. Higher miR-124 expression in tumors—presumably resulting in reduced SB repair and greater sensitivity to chemotherapy—was significantly associated with better survival (left panels of Fig 6) in the subgroup of patients who received chemotherapy. In contrast, patients who did not receive chemotherapy did not show such an association (right panels of Fig 6). The statistical power of this analysis was evaluated using the Cox-proportion hazard model, resulting in an (1-β), i.e. statistical power, of 0.941, which means our sample size was statistically adequate to address this association. Though, in this dataset, the information of the drugs used in chemotherapy is not clearly indicated, the treatment protocols of breast cancer are relatively standardized in individual oncology clinics in different countries. Based on the guideline of NCCN Clinical Practice Guidelines in Oncology, the most common drug used for chemotherapy of breast cancer patients is Adriamycin (Doxorubicin), a drug causing DNA SBs.

## Discussion

Genomic instability is one of the hallmarks of cancer initiation and progression, implying that some cancer cells may have a limited repertoire of DNA repair mechanisms. This weakness can be exploited to specifically kill tumor cells. In addition, many recent efforts have focused on identifying new drug targets, particularly ones that may be involved in DNA repair [21, 30]. For example, PARP1 inhibitors hinder base excision repair of DNA damaged by endogenous or exogenous agents, resulting in accumulation of SBs, which upon conversion to double-strand breaks induce apoptosis of cells deficient in double-strand break repair [38]. Our present study identified miR-124 as a potential intervention agent to target DNA repair genes and pathways, a finding of particular interest and importance in cancer chemotherapy.

The MB-MDA-231 was considered as the typical breast cancer cell line to represent the triple-negative breast cancer [39]. Following initial clue (i.e. the result of Fig 2) suggesting the role of miR-124 in DNA repair based on MDA-MB-231, we purposely only use osteosarcoma cell line, i.e. U-2 OS to explore the mechanisms of miR-124 in DNA SB repair. As mentioned previously, U-2 OS is more suitable for the experiments of DNA repair and response, because that this cell harbors intact *TP53* and *BRCA1* and shows normal DNA repair capacity [30, 31]. In contrast, loss of p53 and BRCA1 in MDA-MB-231 might significantly confound mechanistic experiments in Figs 3 and 4. After the identification of ATMIN and PARP1 as the target of miR-124, we re-used MDA-MB-231 to show restoration of ATMIN and PARP1 expression reverses the DNA repair defect induced by miR-124 overexpression and increases resistance to anti-tumor drugs.

The microRNA Cancer Association Database has shown that miR-124 expression is downregulated in almost all cancers [40]. At the functional level, miR-124 not only decreases cell proliferation and differentiation but also acts as a suppressor of invasion and metastasis [10, 11, 41, 42]. These data suggest a universal tumor suppressor function for miR-124, which is unique among microRNAs in that most play dual roles—as oncogenes or tumor suppressors—in different tumorigenic pathways in different forms of cancer. The findings of the present study suggest an additional function for miR-124 as a tumor suppressor in cancer chemotherapy. In particular, breast cancer patients with high miR-124 expression receiving chemotherapy showed better survival. The exclusively tumor-suppressive function of miR-124 makes it a particularly good candidate to be further developed for use in cancer therapy.

In the present study, we found that miR-124 may be involved in DNA repair by directly targeting ATMIN and PARP1, suggesting that multiple DNA repair pathways are affected by miR-124 and therefore manipulation of miR-124 level/activity may improve the efficacy of chemotherapies that induce DNA damage. ATMIN and PARP1 are involved in multiple aspects of DNA damage response and repair. In the DNA damage response, ATMIN specifically forms Rad51-containing foci in response to DNA-methylating agents [43, 44], and ATMIN ubiquitination by UBR5 is vital for ATM pathway selection and activation [45]. ATMIN is also required during the maturation of B cells and for the repair of DNA SBs that are generated during immunoglobulin class switch recombination [27]. In the present study, repression of ATMIN enhanced the HR repair defect induced by miR-124, and restoration of ATMIN reversed the effect of miR-124 overexpression in breast cancer cells. Therefore, it is intriguing to further speculate which of the multiple roles of ATMIN is specifically affected in breast carcinogenesis. On the other hand, PARP1-mediated processes play a role in oncogenesis, cancer progression, and therapeutic resistance [46, 47]. Various PARP1 inhibitors, some of which are in clinical trials, are being developed for treatment of different cancer types. It is not surprising that the exact mechanisms by which PARP1 inhibitors kill cancer cells remain unknown because PARP1 is a multifunctional protein implicated in various cellular responses to DNA damage ranging from DNA repair and apoptosis to stress signaling, transcriptional modulation, and maintenance of genomic stability [38]. In our study, compared with ATMIN, though PARP1 appears to play a relatively minor role in miR-124-mediated HR, restoration of PARP1 upon miR-124 overexpression led to a significant increase in the SB repair, as detected by comet assay, and the efficacy of ETO and CPT. Further exploration of the specific PARP1-mediated DNA repair mechanisms regulated by miR-124 is needed to develop miR-124 as a cancer therapy.

## Supporting Information

S1 Fig The relationship between miR-124 expression level and the sensitivity of cell to anti-tumor drugs. (A) miR-124 expression level, detected by quantitative RT-PCR, in two stably-transfected clones (miR-124-1 and miR-124-2) and two vector controls (VC1 and VC2) (left panel). These two clones expressing high and low levels of miR-124 were used in clonogenic survival assay (right panel). After treatment of CPT (5 nM, 24 hr), the clone expressing higher level of miR-124 (i.e. miR-124-1) shows lower survival fraction. (B) miR-124 expression level in transiently-transfected clones, which were transfected with different dosages of miR-124 (left panel). The effect of miR-124 to regulate sensitivity to anti-tumor drug is shown after the treatment of CPT (5 nM, 24 hr) (middle panel). In right panel, the sensitivity of cell to different anti-tumor drugs was shown to be affected by cells transfected with different dosages of miR-124. Breast cancer cell line MDA-MB-231 was used in (A) and (B).
(TIF)

S2 Fig Mature miR-124 level in stably miR-124-overexpressing cell. (A) breast cancer cells (MDA-MB-231) stably transfected with the empty lentivirus vector (vector control, VC) or the miR-124-overexpressing construct (miR-124) (B) osteosarcoma cells (U-2 OS) stably transfected with the empty lentivirus vector (vector control, VC) or the miR-124-overexpressing construct (miR-124). miR-124 expression level was determined by quantitative PCR and expressed as the fold change relative to RNU6B. Expression in VC was used as the reference.
(TIF)

S3 Fig Comet assay of miR-124 overexpression (miR-124) and vector control (VC) after treatment with CPT. The result showing that miR-124 overexpression (miR-124) reduces SB repair relative to the vector control (VC) after treatment with CPT.
(TIF)

S4 Fig Restoration of ATMIN and PARP1 level detected by western blotting. (TIF)
