# Supplementary figures and images for: The Effect of MicroRNA-124 Overexpression on Anti-Tumor Drug Sensitivity
Source: PLoS One. 2015 Jun 26;10(6):e0128472. doi: 10.1371/journal.pone.0128472 (PMC4482746; doi:10.1371/journal.pone.0128472)

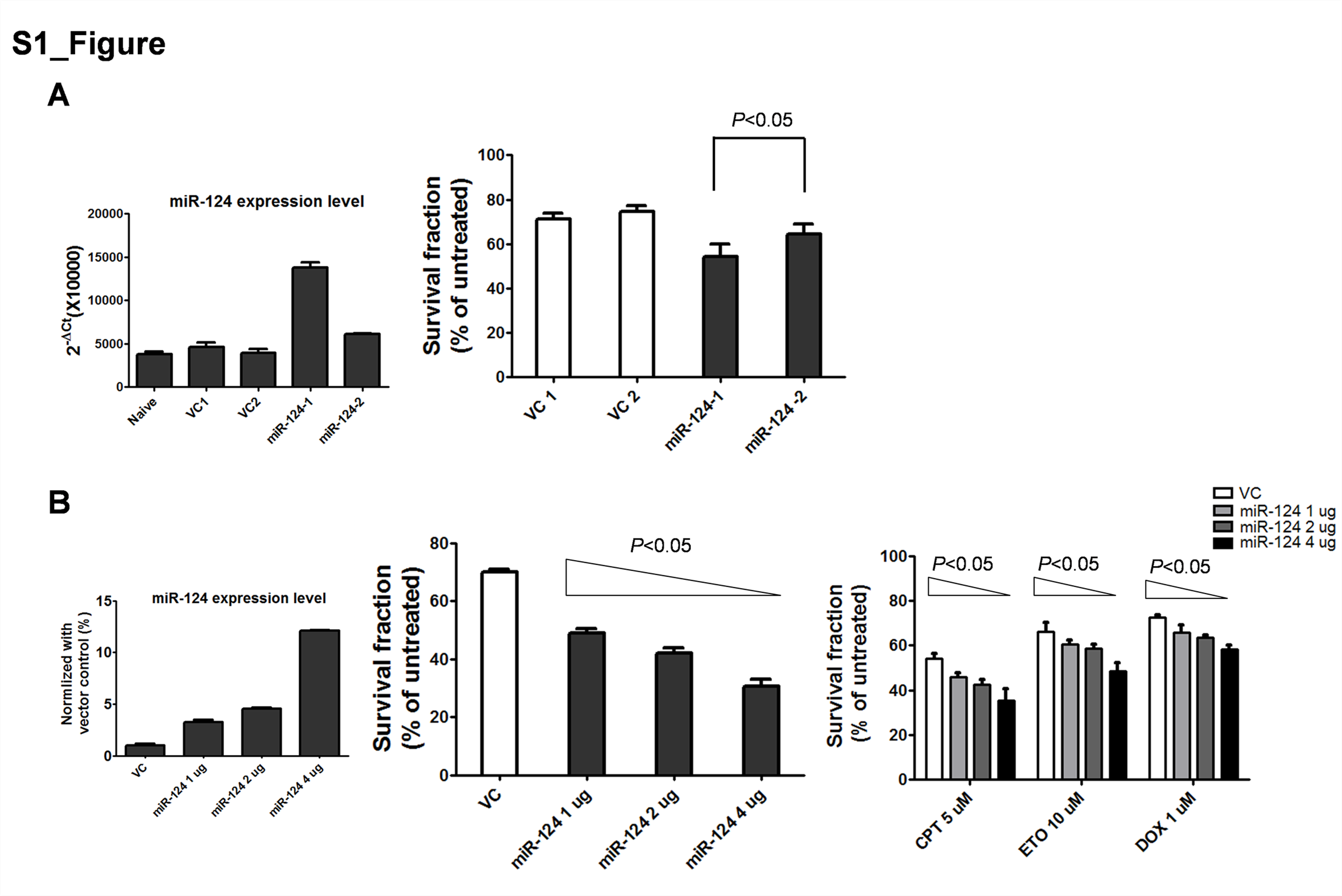

Supplement: S1 Fig — (A) miR-124 expression level, detected by quantitative RT-PCR, in two stably-transfected clones (miR-124-1 and miR-124-2) and two vector controls (VC1 and VC2) (left panel). These two clones expressing high and low levels of miR-124 were used in clonogenic survival assay (right panel). After treatment of CPT (5 nM, 24 hr), the clone expressing higher level of miR-124 (i.e. miR-124-1) shows lower survival fraction. (B) miR-124 expression level in transiently-transfected clones, which were transfected with different dosages of miR-124 (left panel). The effect of miR-124 to regulate sensitivity to anti-tumor drug is shown after the treatment of CPT (5 nM, 24 hr) (middle panel). In right panel, the sensitivity of cell to different anti-tumor drugs was shown to be affected by cells transfected with different dosages of miR-124. Breast cancer cell line MDA-MB-231 was used in (A) and (B). (TIF) [file pone.0128472.s001.tif]

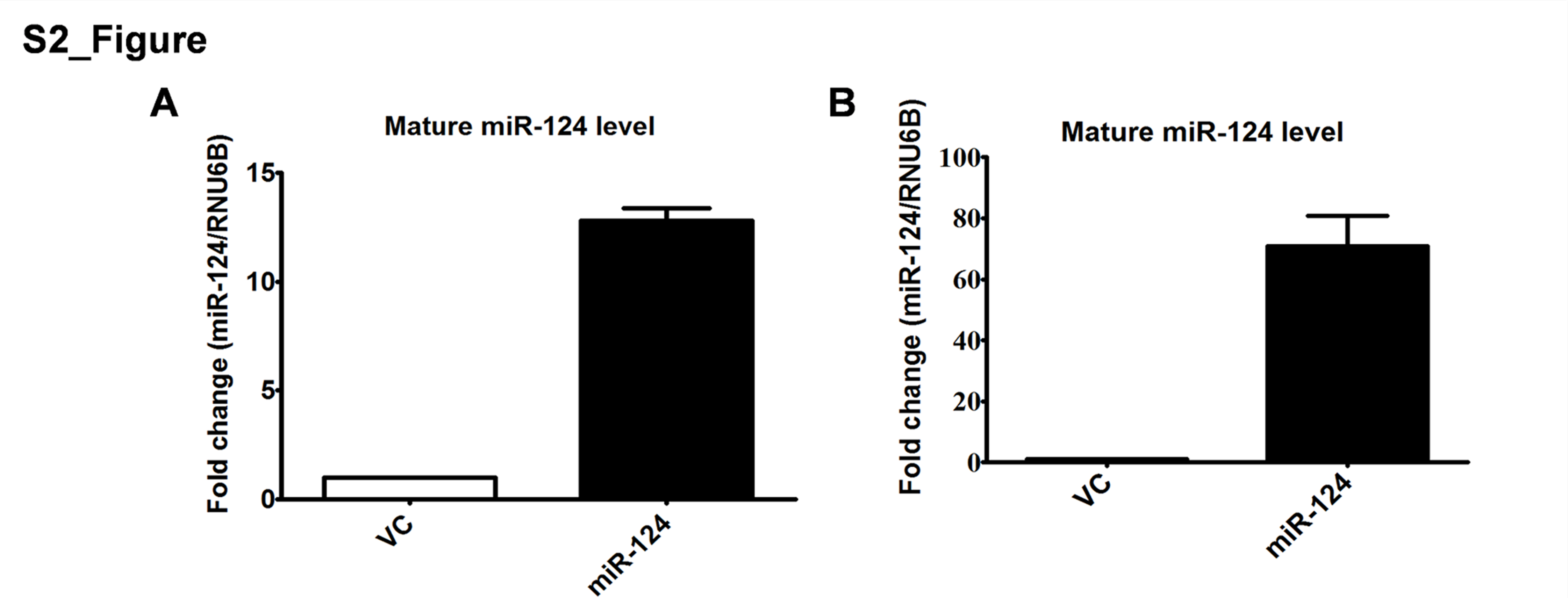

Supplement: S2 Fig — (A) breast cancer cells (MDA-MB-231) stably transfected with the empty lentivirus vector (vector control, VC) or the miR-124-overexpressing construct (miR-124) (B) osteosarcoma cells (U-2 OS) stably transfected with the empty lentivirus vector (vector control, VC) or the miR-124-overexpressing construct (miR-124). miR-124 expression level was determined by quantitative PCR and expressed as the fold change relative to RNU6B. Expression in VC was used as the reference. (TIF) [file pone.0128472.s002.tif]

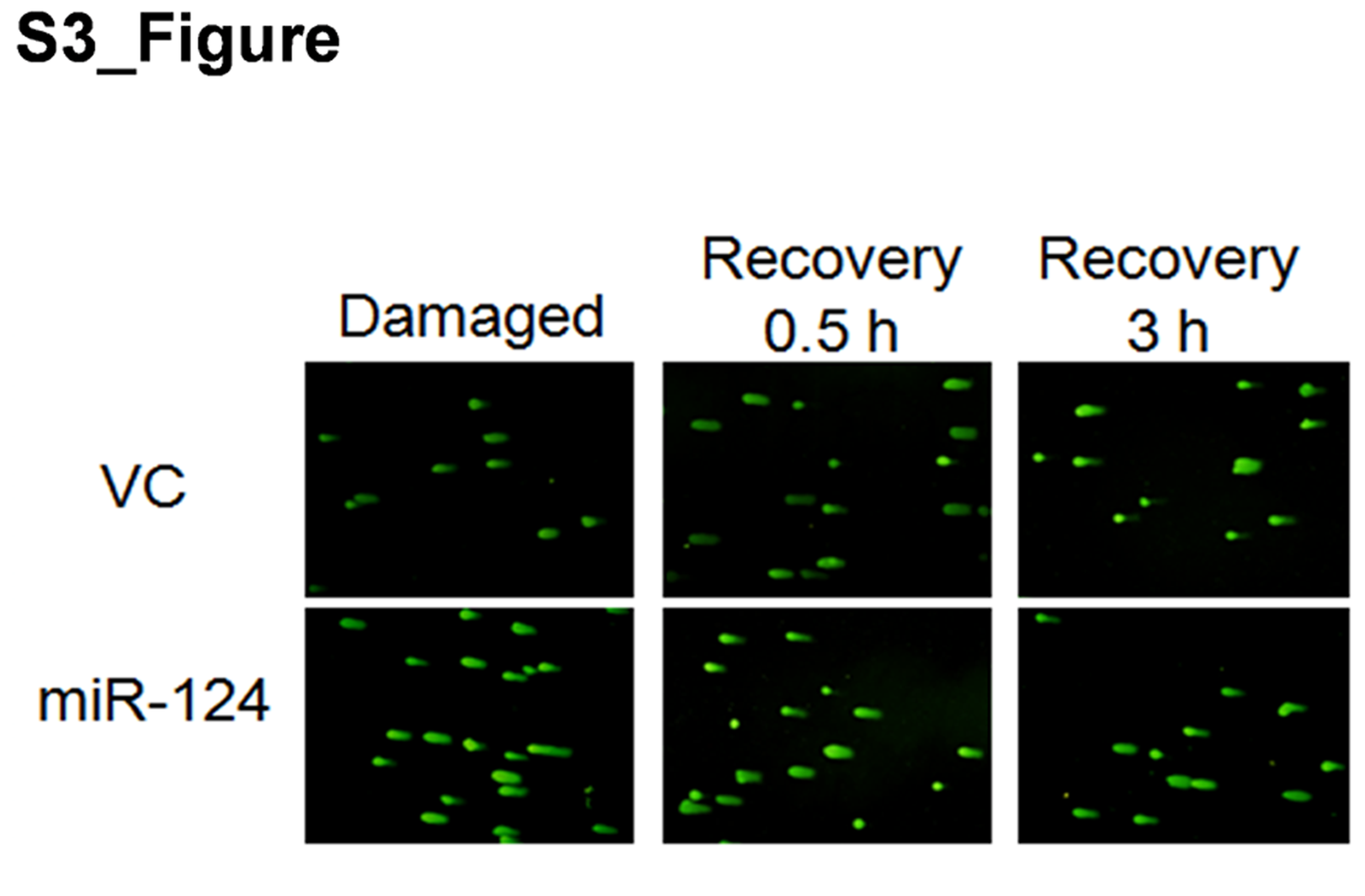

Supplement: S3 Fig — The result showing that miR-124 overexpression (miR-124) reduces SB repair relative to the vector control (VC) after treatment with CPT. (TIF) [file pone.0128472.s003.tif]

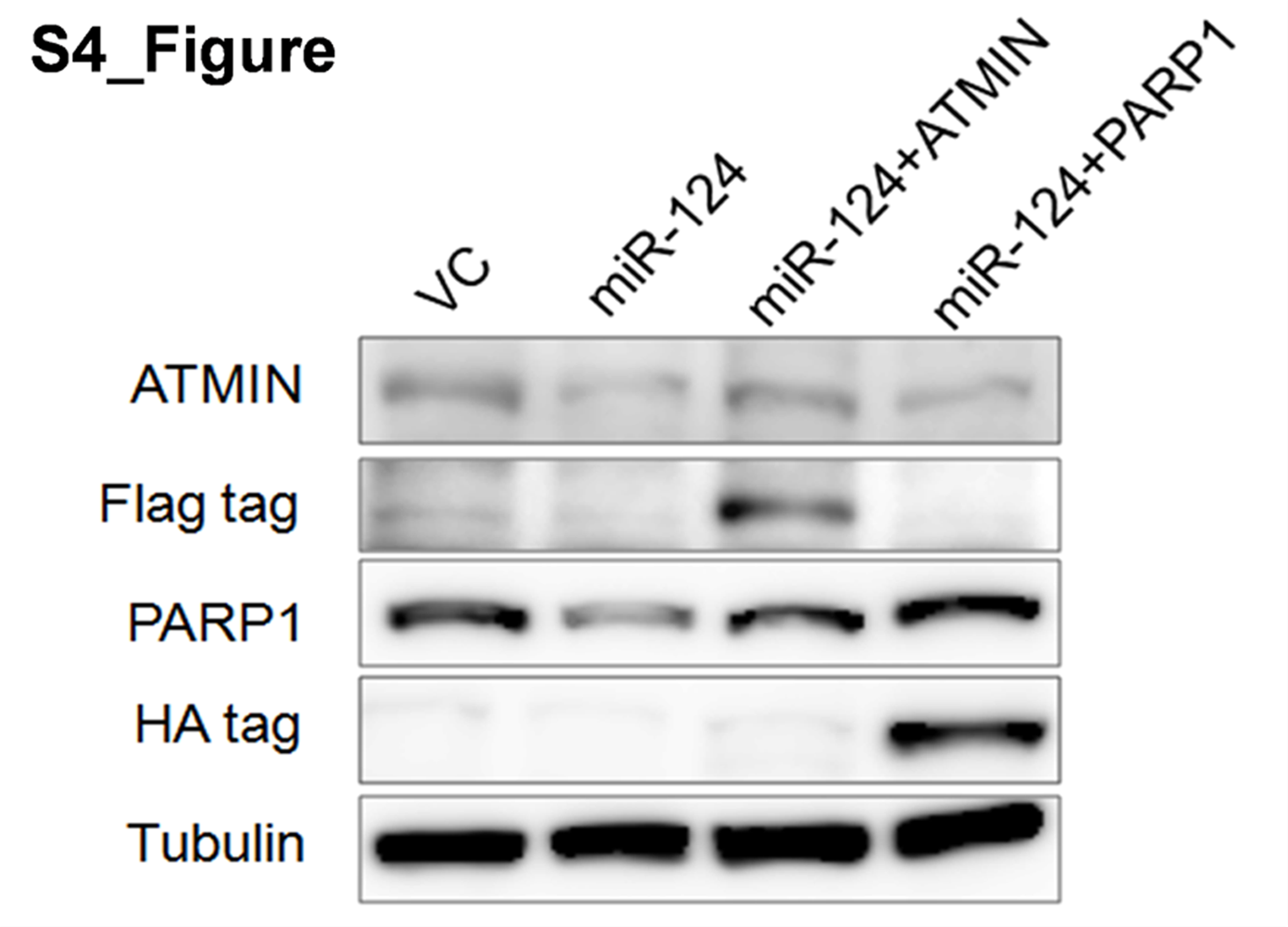

Supplement: S4 Fig — (TIF) [file pone.0128472.s004.tif]
